# Sperm Morphology in Neotropical Primates

**DOI:** 10.3390/ani9100839

**Published:** 2019-10-21

**Authors:** Eliana R. Steinberg, Adrián J. Sestelo, María B. Ceballos, Virginia Wagner, Ana M. Palermo, Marta D. Mudry

**Affiliations:** 1 Grupo de Investigación en Biología Evolutiva (GIBE), EGE, IEGEBA, CONICET, FCEyN, Universidad de Buenos Aires, Pab II, Ciudad Universitaria, Intendente Güiraldes 2160, Ciudad Autónoma de Buenos Aires C1428EGA, Argentina; vickiwagnerr@gmail.com (V.W.); martamudry@yahoo.com.ar (M.D.M.); 2 Laboratorio de Biotecnología Reproductiva, Ecoparque Interactivo (ex ZOO de Buenos Aires), República de la India 3000, Ciudad Autónoma de Buenos Aires 1425, Argentina; asestelo@buenosaires.gob.ar (A.J.S.); mbceballos@buenosaires.gob.ar (M.B.C.)

**Keywords:** sperm morphometry, Platyrrhini, sperm competition

## Abstract

**Simple Summary:**

The spermatozoon is a highly differentiated cell, whose morphology has been affected throughout evolution by selective forces such as the competition between the sperm of rival males (sperm competition) and the joint evolution of male and female reproductive tracts (coevolution). The study of its morphology is important when analyzing the relationships between different species. In this contribution we analyzed new specimens and produced a database with all the spermatozoa dimensions recorded to date, comprising 75 individuals from 20 species and 8 genera, representing 2 families of neotropical primates (Cebidae and Atelidae). After an evolutionary analysis, we observed two different trends for the Cebidae and Atelidae families. Narrower and shorter spermatozoa seem to be the ancestral (oldest) form for Cebidae, with an evolutionary trend toward spermatozoa with wider and larger heads in the derived (younger) species. In Atelidae, on the contrary, narrower heads are observed in the more derived groups. We analyzed these results in the context of sperm competition and mating systems in these groups. More studies are needed to improve our knowledge of the evolution of the spermatozoa in neotropical primates.

**Abstract:**

The morphological and morphometric characterization of spermatozoa has been used as a taxonomic and phylogenetic tool for different species of mammals. We evaluated and compared the sperm morphometry of five neotropical primate species: *Alouatta caraya*, *Ateles belzebuth* and *Ateles chamek* of family Atelidae; and *Cebus cay* (=*Sapajus cay*) and *Cebus nigritus* (=*Sapajus nigritus*) of family Cebidae. After the collection of semen samples, the following parameters were measured on 100 spermatozoa from each specimen: Head Length, Head Width, Acrosome Length, Midpiece Length, Midpiece Width and Tail Length. Considering the available literature on sperm morphometry, we gathered data of 75 individuals, from 20 species, 8 genera and 2 families. These data were superimposed on a phylogeny to infer the possible direction of evolutionary changes. Narrower and shorter spermatozoa seem to be the ancestral form for Cebidae, with a trend toward wider and larger heads in derived groups. The spermatozoa of Atelidae may show an increase in total length and midpiece length. Sperm heads would have become narrower in the more derived groups of *Ateles*. Sperm length may increase in the more derived species in both families. Our results are discussed in the context of sperm competition and sexual selection.

## 1. Introduction

The spermatozoon is a highly differentiated cell, whose morphology and physiology are closely associated with fertilization [1]. Eutherian mammals show a high variation in sperm head morphology [2,3,4,5]. In the orders Rodentia, Pholidota and Edentata, and in two families of the order Primates, Lemuridae and Loridae, the sperm head varies from bearing a hook emerging from the apical end to extensions emerging from its base [3,6]. In this context, sperm morphology and morphometry are regarded as informative variables useful in phylogenetic and taxonomic studies on different mammal species [4,5,7,8]. 

From an evolutionary approach, sperm morphology in mammals has been explained by several adaptive models, including sperm competition and coevolution of male and female reproductive characters [3,9,10,11]. Sperm competition has been proposed to shape many sperm features [3,6,12,13]. For example, sperm with elongated heads may show improved hydrodynamic efficiency by offering lower resistance to the medium (i.e., spermatozoa with narrower heads may swim faster). Likewise, an increase in head volume would influence sperm velocity [12,13,14]. Longer sperm swim faster and are more likely to fertilize ova [12]. Therefore, sperm competition is associated with an increase in total sperm length, which results from an increase in the size of all sperm components [13]. An increase in the flagellum would increase the thrust needed to propel the sperm forward [15], while an increase in midpiece volume would increase the amount of energy to fuel sperm motility [14]. Sperm competition would be related to the species mating system and would be higher in polyandrous species (with multiple male partners) [16], with all these characteristics allowing the spermatozoon to better compete with the sperm of rival males.

In Primates, however, there is little information on sperm morphometry from an evolutionary point of view. In particular, this subject has been poorly studied in neotropical primates (new world primates or Platyrrhini), with only a few species studied to this date [2,9,11,17,18,19,20,21,22,23,24].

To increase the current knowledge of primate sperm morphometry, we provide data of new specimens of five neotropical primate species from two families and three genera: Cebidae, *Cebus cay (=Sapajus cay)* (CCY, Illiger 1815) and *Cebus nigritus (=Sapajus cay)* (CNI, Goldfuss 1809); Atelidae, *Ateles chamek* (ACH, Humboldt 1812), *Ateles belzebuth* (ABE, Geoffroy Saint-Hilaire 1812) and *Alouatta caraya* (ACA, Humboldt 1812). We analyzed the value of the selected morphometric variables for distinguishing among species and compared our results with those reported for other neotropical primates. 

We used biogeographic, morphological and chromosome information from available literature to infer phylogenetic relationships in a “Total Evidence” framework [25], and analyzed all available quantitative data on sperm dimension to shed light on the possible direction of evolutionary changes in sperm morphometry in Platyrrhini. We discussed our findings in the context of sperm competition and sexual selection.

## 2. Materials and Methods 

### 2.1. Analyzed Specimens

Semen was obtained from eight adult males from of neotropical primate species *Cebus cay* (CCY), *Cebus nigritus* (CNI), *Ateles chamek* (ACH), *Ateles belzebuth* (ABE) and *Alouatta caraya* (ACA). For each species, the number of individuals and identification (last four digits of the chip number in brackets) were as follows 3 CCY (D000, ED08 and 4FF6), 1 CNI (991F), 1 ACH (44AF), 1 ABE (C97C) and 2 ACA (C73C and ADD7). Their exact age was unknown, because most of them were rescued from illegal trade and held in captivity, except for CCY D000, who was born in the zoo and was 6 years old at the time of the study. Nevertheless, they were older than 13–14 years, according to a veterinarian’s examination and time spent in captivity. Some males had successfully sired offspring in captivity (CCY ED08, CCY 4FF6, ACA ADD7, ACH 44AF). They were kept at Ecoparque Interactivo (former Buenos Aires Zoo) in heated 3.5 × 3.5 × 5 m enclosures, each of which communicated with outdoor space of the same size with sunlight, soil, plants and height strata. For each species, monkeys were daily fed a diet that satisfies the nutritional requirements of an adult, which was designed by the Nutrition Department. Routine veterinary studies were performed periodically to ensure the health status of the animals. To improve animal welfare, they were subjected to regular veterinarian check-up and behavioral enrichment including social, occupational, physical and sensory stimulation according to their need.

### 2.2. Ethical Note

All experimental protocols complied with the Code of Ethics of Latin American Zoo and Aquarium Association (ALPZA, 2011, available at http://www.waza.org/en/site/conservation/code-of-ethics-and-animal-welfare) and to the American Society of Primatologists (ASP) Principles for the Ethical Treatment of non-human Primates (2 October 2001: available at https://www.asp.org/society/resolutions/EthicalTreatmentOfNonHumanPrimates.cfm). In addition, the study was approved by the “Comisión Institucional para el Cuidado y Uso de Animales de Laboratorio” (CICUAL, or Institutional Commission for the Care and Use of Laboratory Animals) of the Facultad de Ciencias Exactas y Naturales (FCEyN) —Buenos Aires University (http://users.df.uba.ar/zeke/). 

Semen was collected during the routine veterinary evaluation of the specimens. 

### 2.3. Semen Collection

The specimens were sedated indoors with Ketamine (Brouwer 50 mg/mL, Buenos Aires, Argentina at doses of 25 mg/kg IM for CCY and CNI, and 20mg/kg IM for ACA, ACH and ABE). Then, they were transferred to the operating room and placed on a stretcher. The anesthesia was maintained with Isoflurane (Scott Cassara, Buenos. Aires., Argentina, 100% Isoflurane) using a Jackson-Rees circuit with an endotracheal tube N° 3.5 for CCY and CNI and N° 5.0 for ACA, ACH and ABE.

Semen samples were obtained by electro-ejaculation using the methodology of Hernández-López et al. [26], with modifications. Briefly, a probe (12.0 cm in length and 10 mm in diameter—P.T. Electronics, Boring, OR, USA), with three longitudinal electrodes (30 mm), was placed in the rectum. Eighty electrical stimulations were delivered with a 60-Hz sine-wave stimulator (P.T. Electronics, Boring, OR, USA). Stimulations from 2 to 5 V were delivered in sets of 10 each with a repetition of last sets at 6 and 7 V.

After electro-ejaculation, the animals were monitored until complete recovery from anesthesia. Each semen sample was collected in a warmed 7 mL-conical glass tube. The volume of the ejaculate was measured with a micropipette (Gilson’s PIPETMAN Classic) and then transferred to a 1.5 mL-sterile microtube (Axygen, cat: J-MCT-150-C). 

Semen in the studied species coagulates immediately after ejaculation, except for *A. caraya*, where seminal coagulum does not form [27]. In preparing semen smears, it is necessary to release spermatozoa from the highly viscose seminal coagulum. For this purpose, we applied enzymatic treatments for coagulum dissolution, which have proven to maintain sperm morphology in other primates [28,29]. Thus, 5 mg/mL collagenase (B, Roche—Boehringer, Argentina, catalog no. 11088807001) and a solution of 0.25% trypsin in 0.91 mM EDTA (GIBCO, catalog no. 25200-056) were used in 1:1 proportion each. The samples with the enzyme were incubated in a heated bath at 37 °C for 60 min. After that time the enzymatic activity was stopped by adding an aliquot, equal to the enzyme volume, of Fetal Bovine Serum (FBS). Collagenase performed better for disaggregating the *Cebus* spp. coagulum and trypsin worked better in the *Ateles* spp. 

### 2.4. Sperm Morphometric Analysis

In *Alouatta sp.* the smears were made using undiluted semen, while in the other species the solution obtained after the enzymatic treatment was used. At least two semen smears were made for each individual using the feathering technique [30] and stained with buffered Giemsa-differential staining [31]. Sperm motility and quality were assessed in a 10-μL aliquot. The aliquot was placed between a microslide and coverslip and then examined under a phase contrast microscope with a heated stage warmed up to 37 °C. The percentage of motile spermatozoa was recorded. Sperm viability (percentage of live spermatozoa) was assessed by staining aliquots of sperm suspension with nigrosin-eosin (NE), which was prepared as described by Tamuli and Watson [32]. Acrosomal integrity was evaluated on samples fixed in 3% glutaraldehyde buffered solution under a phase-contrast microscope at 400× magnification. For each individual, the following morphometric variables (in μm) were measured on 100 photographed spermatozoa with normal morphology: Head Length (HL), Head Width (HW), Acrosome Length (AL), Midpiece Length (ML), Midpiece Width (MW) and Tail Length (TL). In addition, the following traits were calculated for each spermatozoon: Total Length = HL + TL; Midpiece Volume (MV) = π * (MW/2)^2^ * ML) [11]; Head Ellipticity = HL/HW; Head Elongation = (HL − HW)/(HL + HW) [19,21].

Photomicrographs were taken at 1000× magnification using a Leica DMLB microscope equipped with a Leica DFC 340 FX camera. Linear dimensions were measured using ImageJ 1.47 software (Rasband W.S., NIH, USA) by means of a Wacom Bamboo Pen and Touch tablet device (see Electronic Supplementary Material, Table S1) and were summarized in Table 1.

We created a database from the available literature (Table 2) on sperm morphometry of neotropical primates [2,9,11,17,18,19,20,21,22,23,24], where only the first reference was considered and duplicate entries were removed (e.g., data for *Saimiri sciureus* were first reported by Martin et al. [17] and repeated in [2,9]; then, the latter two were excluded from the analysis). If the number of specimens was missing (such as in [2,11,17]) the record was assumed to belong to a single individual. As a result, we gathered data on 75 individuals, from 20 species, 8 genera and 2 families.

For species with available values of HL and HW, we composed a scale diagram of the head and the anterior midpiece region of the spermatozoon. The sperm morphometric data in the database mentioned above were superimposed on a phylogeny obtained from Dumas and Mazzoleni [33] with modifications to include all the species represented in the literature [34,35,36,37,38,39].

### 2.5. Statistical Analysis

Statistical differences in morphometric variables among the species studied here were tested with one-way ANOVA followed by Bonferroni’s post hoc correction for multiple comparisons. Results were analyzed using Statistica 6.0 software (Statistica, StatSoft Inc., Tuls, OK, USA). The level of significance was set at *P* < 0.05.

## 3. Results

In *Alouatta caraya*, we obtained 50 µL of semen from ACA ADD7 and 70 µL from ACA C73C, both of which had good motility (70 and 50%) and a high percentage of normal spermatozoa (59 and 69%), respectively. *Ateles chamek* yielded 500 µL of liquid fraction, with 50% of sperm motility and 46.8% of normal spermatozoa, plus 1000 µL of seminal coagulum. *Ateles belzebuth* produced 200 µL of liquid fraction, with 25% of sperm motility and 47.5% of normal spermatozoa, plus 4000 µL of seminal coagulum. In *Cebus*, the whole ejaculate coagulated after collection (CCY ED08: 100 µL, CCY 4FF6: 500 µL, CCY D000: 50 µL and CNI 911F: 250 µL), except for CCY D000, who produced 100 µL of liquid fraction with 80% of motility and 67.3% of normal spermatozoa. Prior to the enzymatic treatment, sperm within the coagulum of both *Ateles* and *Cebus* showed less than 10% motility, possibly due to its high viscous consistency. After accomplishing coagulum dissolution, the motility of free-swimming spermatozoa increased slightly (up to 20%), but we obtained satisfactory results of sperm viability (98% for CCY ED08, 52% for CCY 4FF6, 78% for ACH 44AF and 31% for ABE C97C) and an acrosomal integrity higher than 60%, which allowed for morphometric analysis.

**Figure 1:**
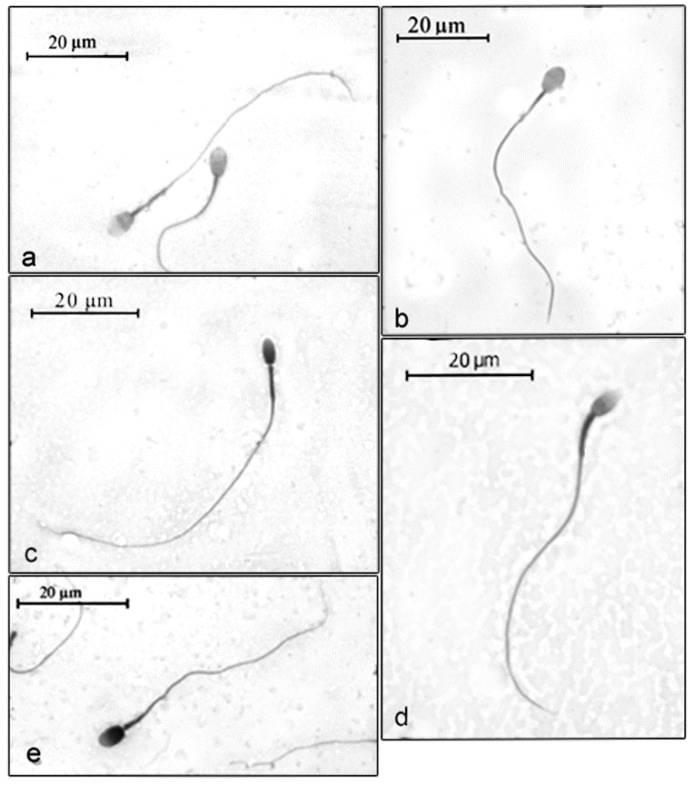
Microphotographs of spermatozoa from the studied neotropical primates. (**a**) *Cebus cay* (=*Sapajus cay*); (**b**) *Cebus nigritus* (=*Sapajus nigritus*); (**c**) *Ateles belzebuth*; (**d**) *Ateles chamek*; (**e**) *Alouatta caraya*.

The spermatozoa of the specimens analyzed in this study showed an oval-rounded head and a central insertion of the tail (Figure 1).

Table 1 shows the Mean (±SD) of morphometric measurements for sperm obtained from the studied neotropical primates.

**Figure 2:**
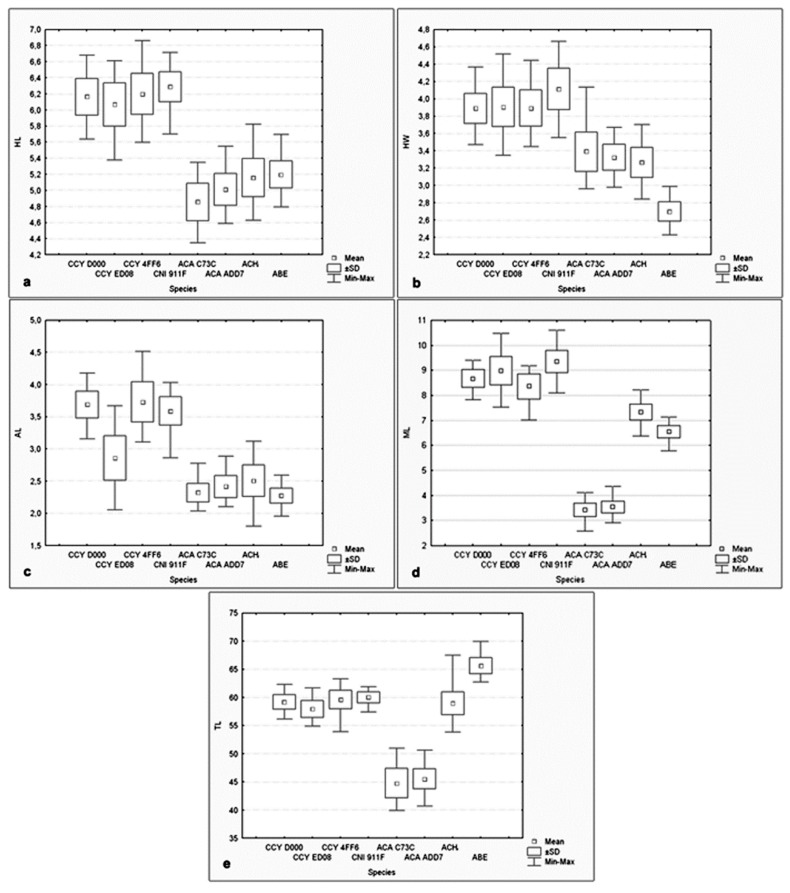
Box plots for the morphometric measurements made on spermatozoa of the studied neotropical primates. (**a**) Head Length (HL); (**b**) Head Width (HW); (**c**) Acrosome Length (AL); (**d**) Midpiece Length (ML); (**e**) Tail Length (TL). The square within the box indicates the mean, the boxes indicates the standard deviation interval (±SD) and the and the ends of the vertical line indicate the maximum and minimum values. *Cebus cay* (CCY, =*Sapajus cay*); *Cebus nigritus* (CNI, =*Sapajus nigritus*); *Ateles belzebuth* (ABE); *Ateles chamek* (ACH); *Alouatta caraya* (ACA).

Most of the measurements differed significantly among species (One-Way ANOVA, *p* < 0.05). In the family Cebidae, no significant differences were found in sperm head length between *C. cay* and *C. nigritus.* In the family Atelidae, no significant differences were observed in head length among *Ateles chamek* and *Ateles belzebuth*; in head width and acrosomal length among *Alouatta caraya* and *Ateles chamek;* and in acrosomal length between *Alouatta caraya* and *Ateles belzebuth*. When comparing species belonging to different families, there were no significant differences in sperm tail length between *Cebus cay* and *Ateles chamek*. The box plots shown in Figure 2 illustrate the distribution of the different morphometric variables obtained for the spermatozoa of the studied neotropical primates.

The comparison between genera revealed that Cebidae had significantly higher values of head length (HL, Figure 2a) and acrosome length (AL, Figure 2c) than did Atelidae (HL: 6.24 ± 0.34 µm vs. 5.26 ± 0.19 µm; AL: 3.72 ± 0.42 µm vs. 2.35 ± 0.32 µm). Within the genus *Cebus*, AL was significantly shorter in ED08 than in the other individuals. Head width (HW, Figure 2b) differed significantly between families, with *Ateles belzebuth* showing the lowest value in Atelidae, while midpiece length (ML, Figure 2d) and tail length (TL, Figure 2e) differed significantly among genera.

In Cebidae, *Cebus cay* showed significantly shorter midpiece length than did *C. nigritus*. In Atelidae, midpiece length and tail length were significantly shorter for *Alouatta caraya* than for *Ateles chamek* and *A. belzebuth*. *A. chamek* had a significantly longer midpiece length than did *A. belzebuth*. In turn, the latter had the narrowest sperm head of all the analyzed species. The analysis of the midpiece volume (see last column in Table 1) indicated that *A. caraya* showed the lowest and *Ateles chamek* the largest value within the family Atelidae. In Cebidae, this variable showed no significant differences between *Cebus cay* and *C. nigritus*.

**Figure 3:**
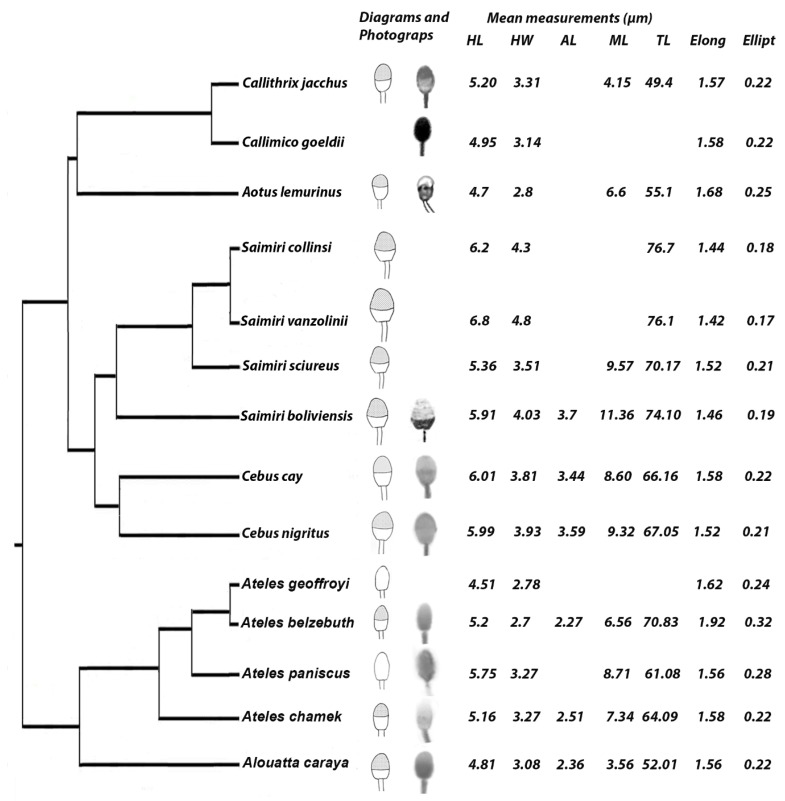
Evolution of sperm morphology and morphometry. Mean sperm measurements were superimposed on a phylogeny of neotropical primates (phylogeny from Dumas and Mazzoleni [33] with modifications [34,35,36,37,38,39]). When available, a scale diagram and/or microphotograph of the sperm head and proximal portion of the midpiece were provided for each species. Only species with data of head length and head width were included. HL: Head Length, HW: Head Width, AL: Acrosome Length, ML: Midpiece Length, TL: Total Length, Ellipt: Ellipticity = (HL/HW), Elong: Elongation = ((HL − HW)/(HL + HW)). *Cebus cay = Sapajus cay; Cebus nigritus = Sapajus nigritus.*

When we compared the results obtained in this study with those published in the literature (Table 2 and Figure 3), we found that, among Cebidae, the genus *Saimiri* had the longer and wider sperm head, with *S. vanzolinii* showing the largest one. The squirrel monkey *Saimiri boliviensis* showed the longest midpiece (11.36 µm), followed by species of the genus *Saguinus* (10.15 µm). In Cebidae, *Callithrix jacchus* showed the shortest midpiece (4.40 µm) and *Saguinus oedipus* showed the shortest total length (43.60 µm).

In the family Atelidae, the genus *Alouatta* exhibited the shortest midpiece of all the Platyrrhini and a total length shorter than that of *Ateles* sp., while *Ateles belzebuth* had the narrowest head and the highest values of elongation (1.92) and ellipticity (0.32).

## 4. Discussion

The present study contributes to the knowledge of sperm morphometry by providing original data from eight specimens of five neotropical primate species scarcely studied. The results obtained in our work are in agreement with those reported in a previous study using principal component analysis (PCA) with the same variables [40]. The sperm morphometric parameters used here allowed for distinguishing among the different genera analyzed. Within Ateles, our study allows us to distinguish between *A. belzebuth* and *A. chamek*, and it is worthwhile mentioning that this is the first report of their sperm morphology and morphometry. Despite the small number of specimens studied here, some sperm head measurements are similar to other previously described for *Ateles*. Compared with measurements reported in the literature, our results were as follows: values of sperm head length were higher for *A. belzebuth, A. paniscus* and *A. chamek* and lower for *A. geoffroyi* [21]. Values of sperm head width were similar for *A. belzebuth* and *A. geoffroyi*, but higher for *A. chamek* and *A. paniscus* (Table 2). Our results for sperm from *Cebus cay*, *C. nigritus* and *A. caraya* are in agreement with those of previous studies applying similar staining techniques ([11,21]; Table IV in [20]).

Regarding the family Cebidae, *C. cay* and *C. nigritus* are very closely related species, naturally distributed in northern Argentina. They are easily distinguishable at the chromosomal level, but their karyotype is highly homologous [41], and hybrids have been obtained in captivity [42]. In this context, it is interesting to highlight that our results did not distinguish between spermatozoa at the interspecific level. Then, it is possible that they share sperm morphometric characteristics involved in sperm-oocyte recognition.

The comparison of sperm measurements between neotropical primate species is a challenging task for many reasons. The small number of specimens so far studied (i.e., 75 individuals from 20 species) is most likely because sperm samples are taken in zoos and breeding centers, since sampling in the wild is a more difficult and expensive endeavor. In addition, even in these facilities, semen collection from sedated animals is not a routine procedure. As a result, studies only include 1 to 5 individuals from each species, as seen in Table 2 and references therein. In addition, studies may differ in terms of the methodology applied for the collection and analysis of sperm morphometric data. Moreover, the wide variation in sperm morphology and morphometry not only between species, but also between individuals within the same species [14,22,24,43,44,45,46,47,48,49,50] makes interspecific comparisons even more difficult. In this sense, the present work makes a useful contribution to our knowledge of the morphometrics of sperm in neotropical primates, shedding light on evolutionary relationships, keeping in mind that new studies in these and other species would bring more light into this very little addressed topic.

The analysis of sperm morphometry from the literature (see Table 2) and from our results (see Figure 3), reveal some interesting evolutionary trends.

In Cebidae, the most basal genera (*Callithrix, Callimico* and *Aotus*) show a spermatozoon with narrower head and reduced total length, and thus it is proposed as the ancestral form. The monogamous genera *Callimico* and *Aotus* have the narrowest sperm heads among the Cebidae (with *Aotus lemurinus* showing the highest elongation and ellipticity values), which is contrary to what would be expected for species with low sperm competition; however, they exhibit the shortest spermatozoa of the family. It will be interesting to perform a more in-depth investigation of the role of sperm morphometry in the evolution of sperm competition in these genera. In the genus *Saimiri,* the more derived species *S. collinsi* and *S. vanzolinii* show higher head length and width, with the latter species having the widest head of all the analyzed Platyrrhini. Thus, in Cebidae, we could propose an evolutionary trend toward larger spermatozoa with wider and larger heads in the more derived species.

Atelidae shows an increase in total length and midpiece length, with *Alouatta caraya* having the shortest and *Ateles belzebuth* the longest values. There are no striking differences in head length between *Alouatta* and *Ateles*, but within *Ateles*, the head becomes narrower (with higher ellipticity and elongation values) in the more derived groups *A. belzebuth* and *A. geoffroyi;* in addition, total length is longer in *A. belzebuth*. Therefore, in Atelidae regarding head morphology the trend would be in favor of narrower heads, in the opposite direction as the trend observed in Cebidae. The total length increases in the more derived species, as observed in Cebidae.

In *Ateles*, increased sperm dimensions and narrower heads could be explained by a hypothesis that morphological, behavioral and physiological differences have evolved to contend with intra-sexual competition. The presence of seminal coagulum would be related to higher intra-sexual competition, because it may serve as a deposit for sperm and a vehicle for sperm transport, turns the vagina milieu into sperm-hostile and blocks fertilization by other males that subsequently copulate with the female [51,52]. The presence of seminal coagulum has been recorded in *A. geoffroyi* [53], *A. chamek* and *A. belzebuth* (this study) but not in in *A. paniscus* [54]. It has been suggested that the absence of seminal coagulum is related to the behavior of mate guarding, present in *A. paniscus* but not in the other species, which involves female surveillance for some days after copulation, to prevent her from mating during the fertile period [55,56]. The evolutionary relationship between seminal coagulation, mating behavior and sperm competition in neotropical primates remains a subject of investigation.

In primates, the sperm midpiece volume has been hypothesized to be larger in polyandrous species, where sperm competition would be higher [11]. Most of the species analyzed in this work are polygamous. In Atelidae, *A. caraya* is polygynandrous [57,58]; and *Ateles* spp. have a fission-fusion mating system [59]. In Cebidae, *C. cay* and *C. nigritus* live in multimale–multifemale polygamous groups [60,61,62]; *Callithrix* spp. and *Saguinus* spp. have variable mating systems that include not only monogamy but also polyandry and polygyny [63]; *Saimiri* spp. are polyandrous [64]. On the other hand *Aotus* spp. and *Callicebus* spp. are monogamous [65,66]. However, for *A. caraya* we obtained a value of midpiece volume that falls within the range of monogamous species postulated in that study [11] (see Table 2) (ACA: 2.6 and 2.3 vs. 3.3 μm^3^ for *Callithrix pygmaea*) [11]. Another exception is provided by *Saguinus midas* and *S. oedipus*, for whom large midpiece volumes were obtained but that have been characterized either as monogamous or polygamous. In considering these contradictory results, it should be taken into account that the midpiece volume has been measured and analyzed in very few neotropical primate species (see Table 2) and that some authors criticize the significantly positive relationship between sperm competition and midpiece volume because of the formula used to calculate the latter variable [13]. The hypothesis linking sperm competition with larger midpiece volume needs to be tested in more neotropical primate species.

The coevolution of male and female genital morphology is another selection pressure acting on spermatozoa of mammals. Certainly, natural sexual selection related to sperm competition and cryptic female choice may have driven the coevolution of oviduct length, testicular size, and sperm morphology [67]. Further studies involving more individuals of different neotropical primate species are needed for a more in-depth analysis of the topics discussed herein. Nonetheless, this work represents a solid step toward a more comprehensive knowledge of this important but scarcely investigated group.

## 5. Conclusions

In this contribution, we provide new data from 5 species of neotropical primates, including the first description of the spermatozoa of individuals of *Ateles chamek* and *A. belzebuth*. We gathered all the data available in the literature and produced a database of sperm morphometric variables on neotropical primates. When superimposing these data on a phylogeny we observed two different trends for the Cebidae and Atelidae families. Narrower and shorter spermatozoa seem to be the ancestral form for Cebidae with an evolutionary trend toward spermatozoa with wider and larger heads in the more derived species. In Atelidae, on the contrary, narrower heads are observed in the more derived groups. In both families the total sperm length increases in the more derived groups. More studies in Platyrrini are needed to improve our knowledge of the evolution of its spermatozoa and its relationship with sperm competition and sexual selection.

## Figures and Tables

**Table 1:** Measurements on spermatozoa of each individual of the analyzed neotropical primate species.

Specimen Identification	Head Length (Mean ± SD)	Head Width (Mean ± SD)	Acrosome Length (Mean ± SD)	Midpiece Length (Mean ± SD)	Midpiece Width (Mean ± SD)	Tail Length (Mean ± SD)	Total Length (Mean ± SD)	Midpiece Volume (Mean ± SD)
CCY D000	6.16 ± 0.23	3.89 ± 0.17	3.69 ± 0.21	8.67 ± 0.36	0.86 ± 0.05	59.19 ± 1.32	65.36 ± 1.37	5.05 ± 0.67
CCY ED08	6.07± 0.27	3.91 ± 0.23	2.89 ± 0.34	8.98 ± 0.56	0.78 ± 0.08	57.94 ± 1.5	64.01 ± 1.58	4.31 ± 0.97
CCY 4FF6	6.20 ± 0.25	3.89 ± 0.21	3.73 ± 0.31	8.36 ± 0.49	0.78 ± 0.06	59.60 ± 1.63	65.80 ± 1.70	4.03 ± 0.71
CNI 911F	6.29 ± 0.19	4.11 ± 0.24	3.59 ± 0.22	9.35 ± 0.44	0.84 ± 0.08	59.98 ± 0.97	66.27 ± 0.10	5.20 ± 1.05
ACA C73C	4.86 ± 0.23	3.39 ± 0.23	2.32 ± 0.14	3.43 ± 0.28	0.99 ± 0.09	44.78 ± 2.63	49.64 ± 2.70	2.65 ± 0.54
ACA ADD7	5.01 ± 0.20	3.32 ± 0.15	2.41 ± 0.17	3.54 ± 0.24	0.90 ± 0.08	45.54 ± 1.76	50.55 ± 1.80	2.29 ± 0.45
ACH 44AF	5.16 ± 0.24	3.27 ± 0.17	2.51 ± 0.25	7.34 ± 0.33	0.97 ± 0.09	58.93 ± 2.01	64.09 ± 2.03	5.42 ± 1.08
ABE C97C	5.20 ± 0.17	2.70 ± 0.11	2.27 ± 0.12	6.56 ± 0.24	0.83 ± 0.06	65.63 ± 1.45	70.83 ± 1.47	3.53 ± 0.55

CCY: *Cebus cay (=Sapajus cay)*, CNI: *Cebus nigritus (=Sapajus nigritus)*, ACA: *Alouatta caraya*, ACH: *Ateles chamek*, ABE: *Ateles belzebuth*. All measurements are expressed in μm, except for midpiece volume which is in μm^3^. Mean ± SD based on 100 spermatozoa per animal.

**Table 2:** Morphometric data on spermatozoa of neotropical primates collected from the literature.

Species	Head Length (µm)	Head Width (µm)	Acrosome Length (µm)	Midpiece Length (µm)	Total Length (µm)	Midpiece Volume (µm^3^)	Ellipticity (HL/HW)	Elongation (HL − HW)/(HL + HW)	Number of Specimens Analyzed	References
**Cebidae familiy**										
*Callithrix jacchus*	5			4.4	50				1	[9]
*C. jacchus*	5	3			50		1.67	0.25	1	[2]
*C. jacchus*	5.6			3.9	48.2	0.6				[11]
*C. jacchus*	5.19	3.46					1. 5	0.2	5	[21]
*C. jacchus*	5.19	3.46					1.5	0.2	4	[24]
*Callithrix jacchus* Mean *	**5.20**	**3.31**		**4.15**	**49.4**		**1.58**	**0.22**		
*C. pygmaea*	4.6			8.5	45.5	3.3			1	[11]
*Callimico goeldii*	4.95	3.14					1.58	0.22	5	[22]
*C. goeldii*	4.95	3.14					1.58	0.22	5	[21]
*Callimico goeldii* Mean *	**4.95**	**3.14**					**1.58**	**0.22**		
*Saguinus midas*	5.3			9.1	45.5	10.2			1	[11]
*S. oedipus*	4.7			1.2	43.6	8.9			1	[11]
*Aotus lemurinus*	4.7	2.8		6.6	55.1		1.68	0.25	3	[18]
*Saimiri boliviensis*	6.1	4.3		10.5	76.8		1.42	0.17	1	[18]
*S. boliviensis*	5.71	3.76	3.7	12.21	71.39		1.52	0.21	2	[20]
*Saimiri boliviensis* Mean *	**5.91**	**4.03**	**3.70**	**11.36**	**74.10**		**1.46**	**0.19**		
*S. sciureus*	5.6			10.1	71.1	6.3			1	[11]
*S. sciureus*	5.11	3.51		9.03	69.24		1.45	0.19	1	[17]
*Saimiri sciureus* Mean *	**5.36**	**3.51**		**9.57**	**70.17**		**1.52**	**0.21**		
*S. collinsi*	6.2	4.3			76.7		1.44	0.18	10	[19]
*S. vanzolinii*	6.8	4.8			76.1		1.42	0.17	2	[19]
*Saimiri sp.* Mean *	**5.92**	**4.13**	**3.70**	**10.46**	**73.56**		**1.43**	**0.18**		
*Cebus nigritus*	5.68	3.75		9.29	67.83		1.51	0.21	1	[20]
*C. nigritus*	6.29	4.11	3.59	9.35	66.27	5.2	1.53	0.21	1	This study
*Cebus nigritus* Mean *	**5.99**	**3.93**	**3.59**	**9.32**	**67.05**		**1.52**	**0.21**		
*Cebus cay (ex C. paraguayanus)*	5.87	3.72		8.53	67.26		1.58	0.22	1	[20]
*C. cay*	6.14	3.90	3.44	8.67	65.06	5.05	1.58	0.22	3	This study
*Cebus cay* Mean *	**6.01**	**3.81**	**3.44**	**8.60**	**66.16**		**1.58**	**0.22**		
*C. albifrons*	9			13.2	67.2	8.4			1	[11]
*C. apella*	8.5			11.6	72.7	4.5			1	[11]
*Cebus sp.* Mean *	**6.47**	**3.88**	**3.49**	**9.37**	**66.91**		**1.67**	**0.25**		
Cebidae Mean *	**5.71**	**3.70**	**3.52**	**8.96**	**61.45**		**1.54**	**0.21**		
**Atelidae family**										
*Alouatta caraya*	5	3.09		3.63	53.93		1.62	0.24	1	[20]
*A. caraya*	4.66	2.94					1.58	0.23	5	[21]
*A. caraya*	4.93	3.35	2.36	3.48	50.09	2.65	1.47	0.19	2	This study
*A. caraya*	4.66	2.94					1.58	0.23	5	[23]
*Alouatta caraya* Mean *	**4.81**	**3.08**	**2.36**	**3.56**	**52.01**		**1.56**	**0.22**		
*Ateles paniscus*	5.11	3.27		7.22	67.26		1.56	0.22	1	[20]
*A. paniscus*	6.4			10.2	54.9	13.2			1	[11]
*Ateles paniscus* Mean *	**5.75**	**3.27**		**8.71**	**61.08**	**13.2**	**1.56**	**0.28**		
*A. chamek*	5.16	3.27	2.51	7.34	64.09	5.42	1.58	0.22	1	This study
*A. belzebuth*	5.2	2.7	2.27	6.56	70.83	3.53	1.92	0.32	1	This study
*A. geoffroyi*	4.51	2.78					1.62	0.24	5	[21]
*Ateles* sp. Mean *	**5.00**	**3.01**	**2.39**	**7.04**	**67.39**		**1.66**	**0.27**		
Atelidae Mean *	**4.91**	**3.08**	**2.38**	**5.29**	**59.38**		**1.65**	**0.25**		
					**Total**				**75**	

*Cebus cay = Sapajus cay; Cebus nigritus = Sapajus nigritus; Cebus apella = Sapajus apella.* * The bolds represent mean value for the species according to the individuals analyzed.

## Supplementary Materials

The following are available online at https://www.mdpi.com/2076-2615/9/10/839/s1, Original dataset containing the measurements of 100 spermatozoa with normal morphology per individual (expressed in µm): Head Length (HL), Head Width (HW), Acrosome Length (AL), Midpiece Length (ML) and Tail Length (TL).
